# High-energy-resolution inelastic X-ray scattering spectrometer at beamline 30-ID of the Advanced Photon Source

**DOI:** 10.1107/S1600577520002854

**Published:** 2020-04-06

**Authors:** Ayman H. Said, Harald Sinn, Thomas S. Toellner, Ercan E. Alp, Thomas Gog, Bogdan M. Leu, Sunil Bean, Ahmet Alatas

**Affiliations:** a Advanced Photon Source, Argonne National Laboratory, Lemont, IL 60439, USA; b European XFEL, Holzkoppel 4, 22869 Schenefeld, Germany; c Department of Physics, Miami University, Oxford, OH 45056, USA

**Keywords:** IXS, HERIX, inelastic, high-resolution

## Abstract

Inelastic X-ray scattering is a powerful and versatile technique for studying lattice dynamics in materials of scientific and technological importance. In this article, the design and capabilities of the momentum-resolved high-energy-resolution inelastic X-ray spectrometer (HERIX) at beamline 30-ID of the Advanced Photon Source are reported. The instrument operates at 23.724 keV and has an energy resolution of 1.3–1.7 meV. It can accommodate momentum transfers of up to 72  nm^−1^, at a typical X-ray flux of 4.5 × 10^9^ photons s^−1^ meV^−1^ at the sample. A suite of *in situ* sample environments are provided, including high pressure, static magnetic fields and uniaxial strains, all at high or cryogenic temperatures.

## Abstract 2

The design and capabilities of the momentum-resolved high-energy-resolution inelastic X-ray spectrometer (HERIX) at beamline 30-ID of the Advanced Photon Source are reported.

## 1. Introduction   

The experimental determination of phonon frequencies and phonon dispersion relations in solids and liquids has been a cornerstone of condensed matter physics ever since the days of Debye (Debye, 1912 ▸). Understanding lattice dynamics provides essential information about the elastic and thermodynamic properties of materials (Brüesch, 1982 ▸; Dove, 1993 ▸; Wallace, 1998 ▸; Srivastava, 1990 ▸). Since the first measurements of phonon dispersion using inelastic X-ray scattering (IXS) (Burkel *et al.*, 1987 ▸; Dorner *et al.*, 1987 ▸), and with the advent of high-brilliance third-generation synchrotron sources, high-efficiency X-ray optics and detectors, high-resolution IXS spectroscopy has advanced significantly. Multiple instruments were built at major facilities such as the ESRF (Krisch & Sette, 2017 ▸), APS (Sinn *et al.*, 2001 ▸), SPring-8 (Baron *et al.*, 2000 ▸; Baron, 2016 ▸) and NSLS-II (Cai *et al.*, 2013 ▸). Nowadays, momentum-resolved IXS with few-millielectronvolt (meV) energy resolution instruments exploit their unique advantages, including large energy-momentum transfers and micrometer-size beam to study new materials under ambient and extreme thermodynamic conditions. IXS has become a powerful tool in studying materials of fundamental and technological importance, spanning a broad spectrum of scientific disciplines from fundamental physics and materials science (Le Tacon *et al.*, 2014 ▸; Stekiel *et al.*, 2019 ▸; Baron *et al.*, 2004 ▸; Uchiyama *et al.*, 2004 ▸; Graf *et al.*, 2008 ▸; Blackburn *et al.*, 2013 ▸; Hahn *et al.*, 2013 ▸; Lebert *et al.*, 2020 ▸) to life science (Liu *et al.*, 2008 ▸; Liu *et al.*, 2005 ▸; Krisch *et al.*, 2006 ▸) and geophysics (Kamada *et al.*, 2019 ▸; Finkelstein *et al.*, 2018 ▸; Mao *et al.*, 2012 ▸; Ohtani *et al.*, 2013 ▸; Fiquet *et al.*, 2001 ▸; Takahashi *et al.*, 2019 ▸).

Recently, IXS has been used to study new emerging novel materials such as thermoelectric and topological materials. IXS studies have led to better understanding of the role of lattice dynamics in superionic crystals (Niedziela *et al.*, 2019 ▸), the observation of intrinsic localized modes (Manley *et al.*, 2019 ▸) and double-Weyl phonons (Miao *et al.*, 2018 ▸).

The high-energy-resolution inelastic X-ray spectrometer (HERIX) is a state-of-the-art spectrometer that provides: (i) high resolving power 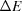/*E* ≃ 6 × 10^−8^ at *E* = 23.724 keV; (ii) micro-focused beam, 35 µm × 15 µm (H × V); (iii) nine analyzers with an angular (momentum) separation of 0.95° (2 nm^−1^) between two adjacent analyzers; and (iv) access to large momentum transfer, *Q*_max_ = 72 nm^−1^. Combining a wide range of momentum transfers with a multi-analyzer setup makes measurements of phonon density of state (DOS) from powder and polycrystalline samples more efficient (Manley *et al.*, 2012 ▸; Bosak & Krisch, 2005 ▸).

The design of the HERIX instrument is based on an angle-tuned in-line high-resolution monochromator (HRM) (Toellner *et al.*, 2006 ▸; Toellner, 2000 ▸), where the incoming and outgoing beams propagate in the same direction. Other high-resolution inelastic X-ray scattering beamlines at the ESRF (Verbeni *et al.*, 1996 ▸; Sette *et al.*, 1996 ▸) and SPring-8 (Baron *et al.*, 2000 ▸; 2001 ▸; Baron, 2016 ▸) use backscattering high-resolution monochromators. For the in-line HRM the energy is scanned by tuning the Bragg angle of the crystals, whereas for the backscattering monochromator the energy is scanned by changing the temperature of the crystal.

In this paper, the design, overall layout and optical components of the HERIX spectrometer are described. The main specifications of the instrument are summarized in Table 1 ▸. The initial commissioning of the beamline was completed in 2008. In 2014, a significant upgrade was performed with the installation of two new short-period undulators. As a result, incident flux was increased by a factor of two, and the stability of the instrument was greatly enhanced due to the lower heat load generated by the short-period undulators.

## 2. Beamline design and optical components layout   

**Figure 1:**
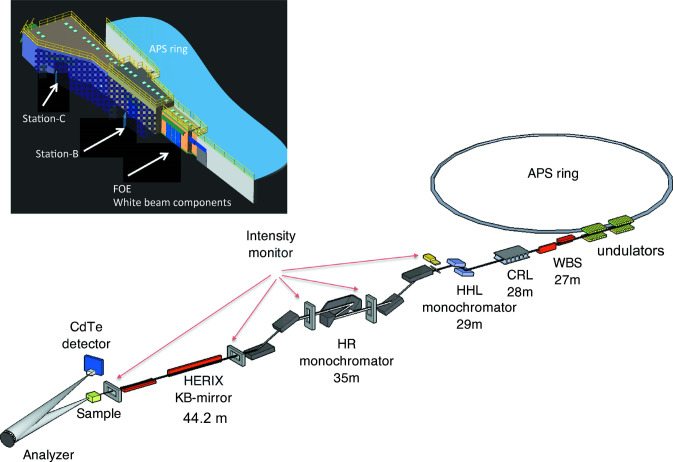
Schematic for the 30-ID beamline layout.

The optical layout of the HERIX spectrometer and the white beam components are shown in Fig. 1 ▸. There are three stations at Sector 30 of the Advanced Photon Source:

(i) Station-A (FOE): contains white beam components and high-heat-load monochromator (HHLM).

(ii) Station-B: houses the high-resolution monochromator (HRM).

(iii) Station-C: contains the actual spectrometer, including focusing optics, sample environments and analyzer systems.

In this section, the main components of the beamline are described.

### 2.1. Source   

**Figure 2:**
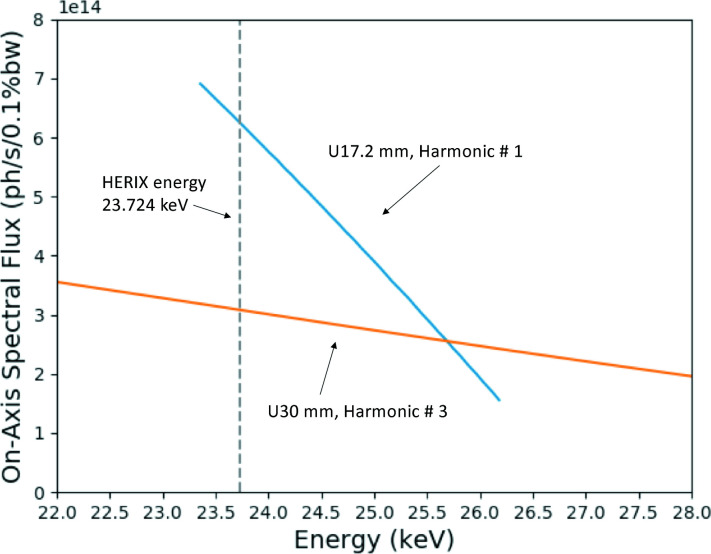
Calculated on-axis spectral flux in 0.4 mm (V) × 1.8 mm (H) aperture at 27.5 m as a function of X-ray energy delivered by the undulators. The blue curve represents the 17.2 mm-period undulator (currently in use at Sector 30) and the orange curve represents the 30 mm-period undulator (used between 2008 and 2014). The calculations were performed for the APS default lattice 2.51 nm rad, coupling 1.5%, 7.0 GeV and 100 mA. The calculated gain in flux at *E* = 23.724 keV is a factor of two.

The X-ray source consists of two undulators optimized to deliver the highest spectral flux at *E* = 23.724 keV in the first harmonic. The length of each device is 2.4 m (total length = 4.8 m), and the magnetic period is 17.2 mm. Fig. 2 ▸ shows the calculated maximum on-axis spectral flux of the undulators for both the current undulator and the original 30 mm-period undulators which were used between 2008 and 2014. A factor of two in flux was realized due to the installation of the short-period undulators in 2014.

### 2.2. High-heat-load monochromator   

The X-ray beam generated by the undulators has a broad spectral distribution with sufficient power (∼kW) to damage spectrometer components. Therefore, reducing the heat load seen by the high-resolution monochromator is critical. The high-heat-load (HHL) monochromator is a water-cooled diamond (111) double-crystal monochromator (DCM) in a non-dispersive (+,−) configuration. The measured energy bandwidth after the HHL monochromator is Δ*E* = 1.6 eV at *E* = 23.724 keV. The mechanics and the vacuum chamber were manufactured by Kohzu Precision Co. Ltd (https://www.kohzuprecision.com/i/). The diamond crystals, 0.3 mm × 4 mm × 6 mm in size, were synthesized by the Technological Institute for Superhard and Novel Carbon Materials (Troitsk, Russia) (Polyakov *et al.*, 2011 ▸; Blank *et al.*, 2007 ▸).

**Figure 3:**
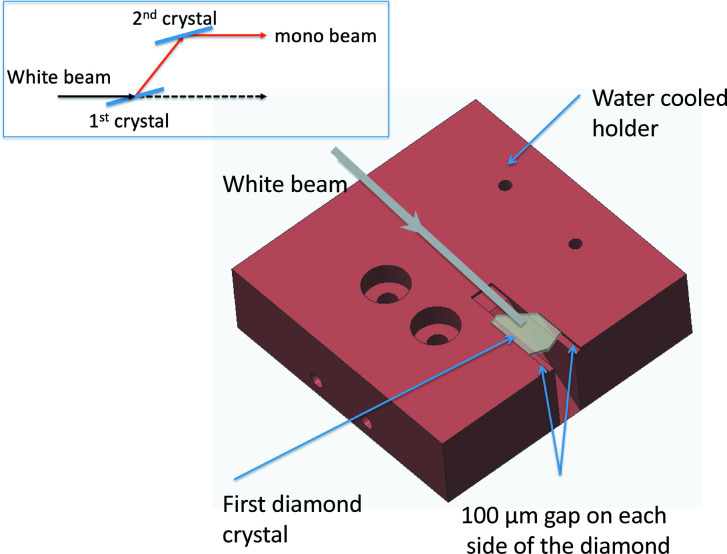
The mount for the first diamond crystal inside the HHL monochromator. The crystal holder and heat exchanger (not shown in the schematic) are made of copper and coated with Ni to protect from GaIn eutectic. The top-left inset is a schematic of the DCM crystal configuration.

Diamond has been used for many years for high-heat-load applications at synchrotron radiation facilities because of its superior thermal conductivity, small thermal expansion and low X-ray absorption. While these characteristics are compelling, the performance of the HHL monochromator critically depends on the quality of the crystal and their strain-free mounting. A custom holder was machined to precisely fit the shape of the first diamond crystal with a small tolerance allowing for thermal expansion. The holder is shown in Fig. 3 ▸. The crystal floats on a thin layer of liquid metal eutectic of gallium–indium (EGaIn) providing excellent thermal conduction between diamond and the holder. No mechanical clamps are used. The crystal is solely held in place by gravity and surface tension.

### 2.3. High-resolution monochromator   

**Figure 4:**
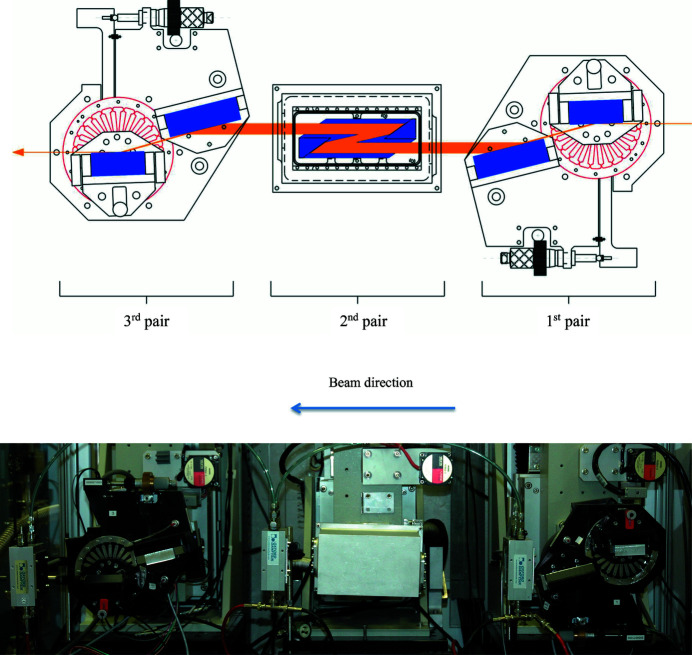
Top: schematic for the six-reflection high-resolution monochromator, which shows the change in beam size (orange lines) as it passes throughout the crystals. Bottom: photograph of the HERIX monochromator.

To further reduce the energy bandwidth of the incident beam to a meV level, a high-resolution monochromator is used (Toellner *et al.*, 2006 ▸). It consists of a sequence of six silicon crystal reflections as shown in Fig. 4 ▸. The first and third pairs of crystals are coupled with a weak-link mechanical joint (Shu *et al.*, 2001 ▸) to allow the rotation of one crystal with respect to the other. Using asymmetric Si(311) crystals, the first pair collimates the beam to a degree that allows the second pair to accept the full beam, thereby increasing the efficiency (Kikuta & Kohra, 1970 ▸). The second pair of crystals is fabricated from a monolithic channel-cut Si(15119), and cooled down to 123 K using a low-vibration custom-made helium-flow cryostat (Toellner *et al.*, 2006 ▸). This temperature corresponds to the zero-thermal-expansion temperature in silicon (Touloukian *et al.*, 1977 ▸), and minimizes the *d*-spacing variation due to any temperature gradient or fluctuation. The monolithic channel-cut is surrounded by a helium exchange gas for better thermal stability. The thermal stability of Si(15119) is better than 2 mK.

The Si(15119) channel-cut monochromatizes the beam to 1 meV energy bandwidth. The size and divergence of the beam after the second pair are restored to their original values using the third pair of Si(220) crystals. During operation, the energy scans (up to ±200 meV) are performed by rotating only the second pair. An angular change of 0.405 µrad corresponds to an energy change of 1 meV. A high-resolution rotation stage, a Kohzu KTG-15 goniometer with 25 nrad resolution, is used for scanning. The design and resolution function are described in full detail elsewhere (Toellner *et al.*, 2011 ▸).

### 2.4. Focusing mirror system   

The ability to focus an X-ray beam to a few micrometers using highly efficient mirrors at a third-generation synchrotron source is of great advantage for X-ray spectroscopy. Particularly for the growing field of high-pressure research, it enables the measurement of micrometer-sized samples. A 12-element bimorph Kirkpatrick–Baez mirror system is installed in the C-station (44.2 m away from the source) to focus the beam at the sample position (46 m from the source). The lengths of the horizontal and vertical mirrors are 900 mm and 450 mm, respectively. Both mirrors have two sets of coatings: palladium (Pd) and platinum (Pt), with Pt having a higher reflectivity at 23.724 keV. The typical horizontal and vertical spot sizes are 35 µm and 15 µm, respectively. The efficiency of the mirror system is within 75–80%. Both mirrors are operated in a vacuum. The horizontal divergence at the sample is 0.9 mrad, which corresponds to a momentum resolution of about 0.017 nm^−1^ at *E* = 23.724 keV.

### 2.5. HERIX analyzer system   

The energy resolution of a crystal analyzer is the convolution of two parts: (i) intrinsic energy resolution of the analyzer crystal, 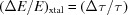, where τ is the length of a reciprocal lattice vector and 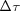 is the extension of the reciprocal lattice point parallel to τ (Authier, 2001 ▸; Burkel *et al.*, 1987 ▸; Baron, 2016 ▸), (ii) geometrical contribution ,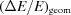 = cot(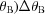, where 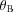 is the Bragg angle and 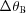 is the variation of the Bragg angle due to the beam divergence and geometrical constraints. While the geometrical contribution vanishes at 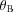 = 90° corresponding to exact backscattering geometry, this is not a practical solution because the sample and detector cannot overlap. However, the geometrical contribution can be minimized by working near backscattering geometry, which in turn puts many constraints on the design of an IXS spectrometer, including a long scattering arm and small separation between the sample and the detector, as will be shown below.

**Figure 5:**
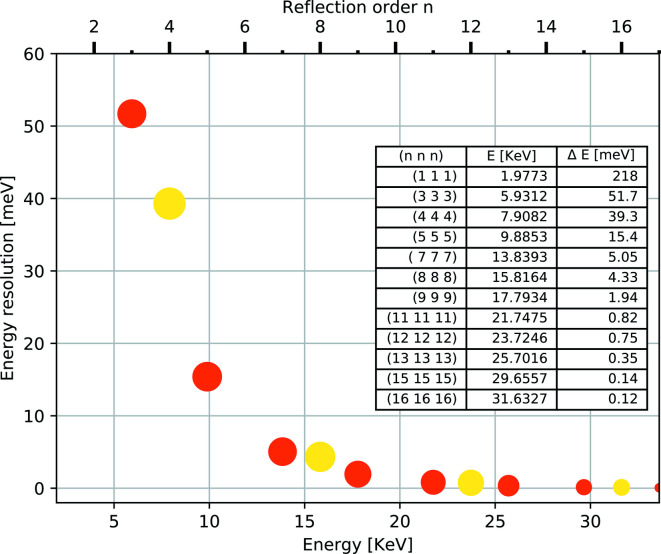
Energy resolution as a function of incident energy and reflection order in Si(*nnn*) at 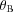 = 90° (Shvyd’ko, 2004 ▸). The solid red circles are odd reflections and the yellow ones are even reflections. The size of the circle represents the relative reflectivity. The even reflections have slightly worse energy resolution compared with the odd ones.

**Figure 6:**
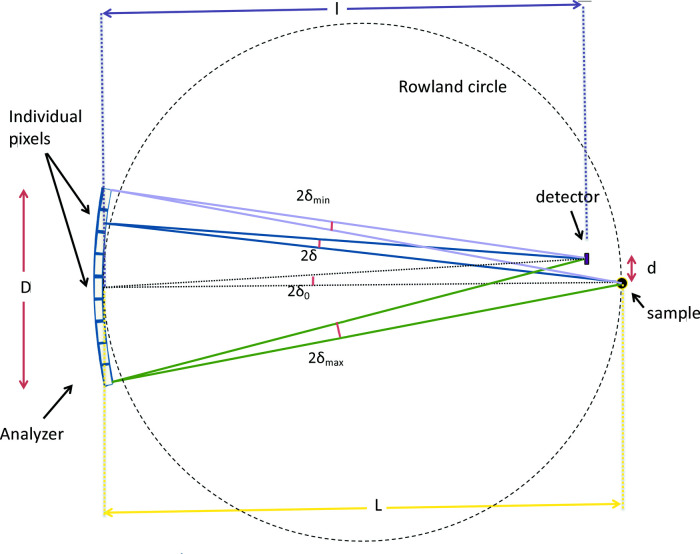
Schematic for the sample, analyzer and detector geometry. *l * is the distance between the detector and analyzer (8910 mm). *L* is the distance between the sample and analyzer (9090 mm). *D* is the vertical opening of the analyzer (100 mm). *d* is the vertical distance between the sample and the detector (3.5 mm). δ_0_ is the angular deviation of the Bragg angle from 90°.

The choice of working energy for a non-resonant IXS spectrometer is determined by the backscattering energy of the analyzer crystal, which in turn depends on the desired energy resolution. As shown in Fig. 5 ▸, a higher energy resolution requires higher X-ray energy to achieve a near-backscattering geometry to minimize the geometrical energy broadening and allows for higher angular acceptance (Graeff & Materlik, 1982 ▸; Shvyd’ko, 2004 ▸). The analyzer crystal for an IXS spectrometer operates typically in Rowland-circle geometry to allow focusing and energy selectivity of the scattered radiation from the sample. However, the closer it is to backscattering, the more difficult it is to use the exact Rowland geometry (Burkel *et al.*, 1987 ▸). As a result, working slightly off the Rowland circle becomes inevitable to allow for space around the sample to accommodate different sample environments. This results in an extra geometrical contribution to the resolution function that is called the demagnification contribution 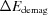 (Burkel *et al.*, 1987 ▸; Sinn, 2001 ▸),
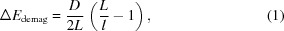
where *L* is the distance between the sample and analyzer (9090 mm), *l* is the distance between the detector and analyzer (8910 mm) and *D* is the vertical size of the analyzer (100 mm) as shown in Fig. 6 ▸. The distance between sample and detector, 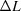 = 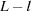 (180 mm), has to be kept as small as possible to avoid degradation in the energy resolution because of the demagnification contribution. The angular deviation from backscattering, 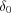 (∼0.2 mrad), also shown in Fig. 6 ▸, is proportional to *d* and 1/*L*, where *d* is the vertical distance between the sample and the detector, and *L* has to be large to achieve higher energy resolution where 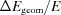 ≃ 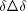. The value of 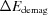 is 0.5 meV. The other geometrical contribution to the resolution function is related to the pixel size (∼1 mm × 1 mm) of the analyzer (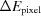 ≃ 0.5 meV) as described in detail elsewhere (Sinn, 2001 ▸). The beam size contribution to the resolution function is negligible (on the order of 0.008 meV).

The other advantage of working near backscattering is the large angular acceptance (Shvyd’ko, 2004 ▸). This is crucial for the spectrometer efficiency in order to accept highly divergent radiation scattered from the sample.

#### 2.5.1. Vacuum chamber of the multi-analyzer arm   

**Figure 7:**
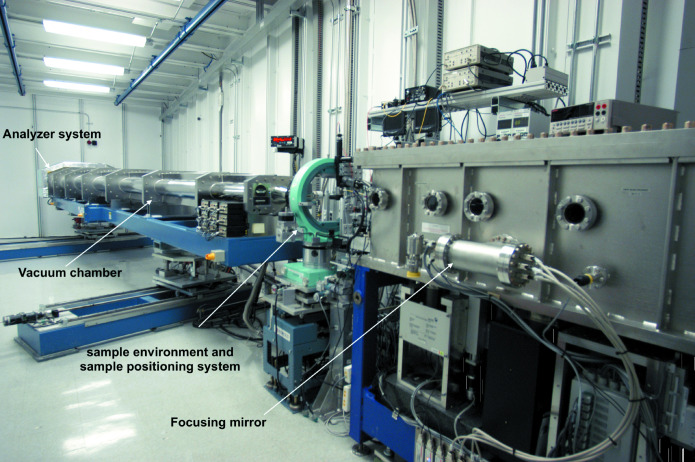
Photograph of the C-station components including focusing mirror system, sample positioning system and analyzer system.

The vacuum chamber is designed to accommodate the flight path of scattered radiation from a sample to nine separated crystal analyzers (analyzers are not in vacuum). The nine analyzers are placed in the horizontal scattering plane. The chamber has the possibility of expanding up to 21 analyzers below and above the current row of analyzers. A photograph of the vacuum chamber, sample table and mirror system in the C-station is shown in Fig. 7 ▸.

#### 2.5.2. Analyzers   

Analyzers are where the energy selectivity of the inelastic X-ray scattering takes place (Masciovecchio *et al.*, 1996 ▸; Sinn *et al.*, 2002 ▸; Verbeni *et al.*, 2005 ▸; Said *et al.*, 2011 ▸). The analyzers are fabricated from Si(121212) crystals and operate at a Bragg angle of 89.98°. The intrinsic energy resolution of the Si(121212) analyzer crystals is 0.75 meV. The solid angle acceptance of the fully open analyzer is 0.095 sterad. Each analyzer consists of 7000–10000 sub-millimeter single-crystal silicon pixels mounted on a spherical lens with radius *R*, where *R* = 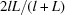 = 8999 mm. The silicon pixels have to be strain-free and cut from a high-quality wafer. The wafers are sliced from a high-resistivity ingot, >50 kΩ cm (Said *et al.*, 2011 ▸).

The angular (momentum) separation between two adjacent analyzers is 0.95° (*Q* = 2 nm^−1^ at *E* = 23.724 keV). Each analyzer is mounted on a motorized Newport 600A-4R stage to allow the adjustment of both 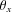 (pitch) and 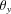 (yaw) angles. The *Q* resolution of the analyzers can be changed using motorized circular apertures, Standa 8MID98-4-H (http://www.standa.lt). The *Q*-resolution range is 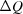 = ±(0.1–0.7) nm^−1^.

The thermal stability of the analyzers is critical for high-resolution spectroscopy. Any temperature change on the analyzer crystal will result in a shift of the energy of the backscattered beam. Also, any temperature gradient within the analyzer will degrade the energy resolution. A change in the analyzer temperature by 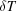 causes the energy of the diffracted beam from the analyzer to change by 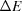, which is given by
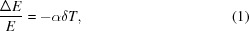
where α = (2.581 ± 0.002) × 10^−6^ K^−1^ is the linear thermal expansion coefficient of silicon at room temperature (Bergamin *et al.*, 1997 ▸).

**Figure 8:**
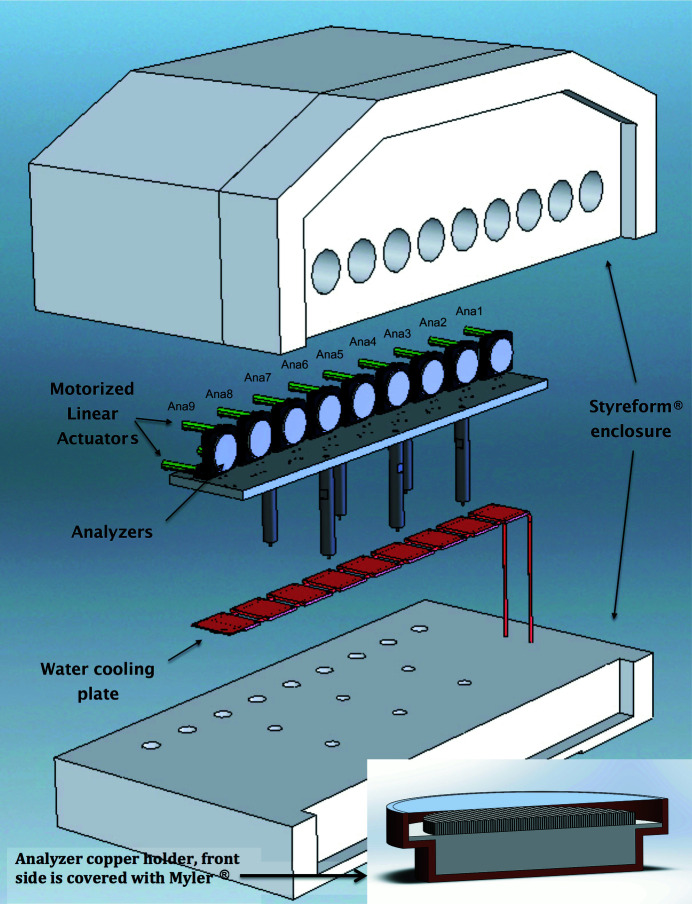
Schematic for the analyzer system including enclosure.

The temperature stability for the analyzers is achieved via thermal isolation and active temperature control. For thermal isolation, a custom-made thick Styrofoam^®^ enclosure is used, as shown in Fig. 8 ▸. Each analyzer is mounted inside a copper cylindrical holder; see the lower right corner of Fig. 8 ▸. The front opening of the copper holder is sealed with an aluminized Mylar^®^. The analyzers and motorized stages are mounted on a temperature-stabilized aluminium base plate. The energy stability depends on the thermal stability of the analyzer as well as the thermal and mechanical stability of the HRM. The temperature stability of the analyzers is around 1–2 mK (0.063–0.13 meV) over 2 h and up to 5 mK (0.31 meV) over 24 h. The large energy drift happens during the liquid-nitrogen filling for the HRM, which is normally done during the first day of the experiment. The energy drift after the liquid-nitrogen filling can go up to 1.5 meV, and instability lasts for a few hours. In order to overcome issues with energy drift, energy scans always include the elastic peak as a marker for the zero- energy-transfer point of the inelastic spectrum.

#### 2.5.3. Detectors and collimator   

Backscattering geometry puts stringent constraints on the integration of the detector into the spectrometer and the necessity for a custom-designed detector system. The sensors need to work in a vacuum, have low electronic noise (less than a few mHz), and high efficiency at *E* = 23.724 keV. The original design of the HERIX spectrometer included nine single-element detectors, which are being replaced with a new detector concept, consisting of two separate, modified CdTe Pilatus3 area detectors by Dectris. They were designed to provide detecting areas above and below the scattering plane. Only the top half of this system was successfully implemented and tested thus far, while the bottom portion will be installed in the near future. The sensors and electronics are water-cooled, the thickness of the CdTe sensor is 1 mm, leading to 100% efficiency at 23.724 keV. The active area is 83.8 mm × 33.5 mm and the pixel size is 0.172 mm × 0.172 mm. While the active area of the detector is large, only a small part of it is used, which is on the edge of the sensor, 3 mm × 33.5 mm.

Position-sensitive detectors are used in inelastic scattering spectroscopy to improve the energy resolution (Huotari *et al.*, 2005 ▸; Shvyd’ko *et al.*, 2013 ▸). Experimentally there was no immediate improvement but it offers the opportunity in the future to reduce the length of the arm while maintaining the same energy resolution. One other advantage of the position-sensitive detector at Sector 30 is that it allows for the characterization of the focus of the analyzer.

**Figure 9:**
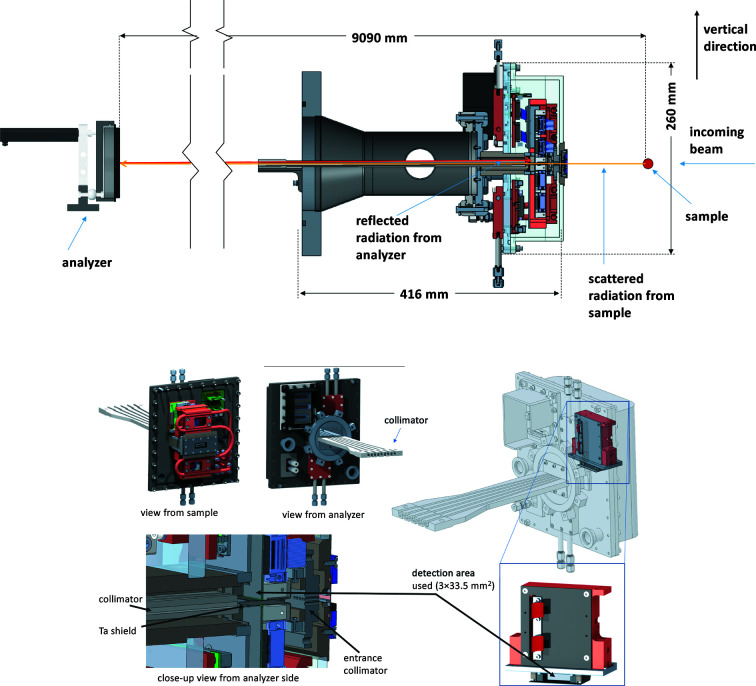
Top: vertical cross section of the HERIX arm, detector and analyzer. Bottom: schematic of the new area detector assembly and collimator.

The size of the focus generated by the analyzers on the detector varies and depends on their figure error. There is potential for contamination due to crosstalk which was experimentally found to be less than 0.5%. To prevent crosstalk, a collimator of 425 mm length was installed at a distance of 15 mm from the detector. A schematic of the collimator is shown in Fig. 9 ▸.

## 3. Resolution function   

**Figure 10:**
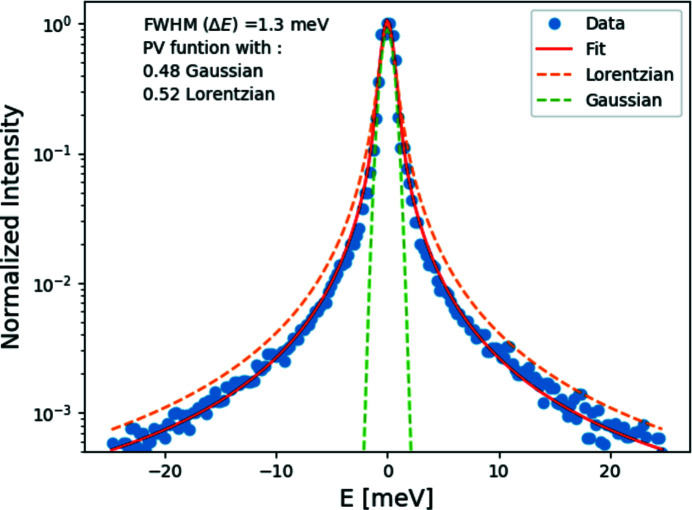
Measured resolution function. The blue solid circles represent the data and the red line represents the pseudo-Voigt fit.

The resolution function is obtained by measuring the elastic scattering from a Plexiglas^®^ sample (10 mm-thick) at room temperature. The elastic scattering is collected at 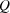 = 10 nm^−1^, which corresponds to the maximum of the structure factor. The measured resolution function and efficiency of the analyzers vary slightly from one analyzer to another. This is due to the slight difference in figure error and temperature gradient on the analyzers, as explained in §2.5.2. The resolution function can be fit with a pseudo-Voigt function. A measured resolution function is shown in Fig. 10 ▸, with an overall energy resolution of 1.3 meV full width at half-maximum (FWHM). The resolution for other analyzers ranges between 1.3 and 1.7 meV. The energy resolution could change by 0.05–0.1 meV due to any temperature gradient on the analyzer.

## 4. X-ray diffraction and thermal diffuse scattering capabilities   

The beamline is equipped with a pixelated area detector that enables diffraction measurements without energy resolution. The area detector is used for aligning samples and determining the change in density in materials as a function of pressure. The sensor is made of amorphous silicon by Perkin-Elmer (https://www.perkinelmer.com; Detector series: XRD 0822). The pixel size is 200 µm × 200 µm, and the active area is 204.8 mm × 204.8 mm. A photograph of the setup at the sample postion is shown in Fig. 12.

As an auxiliary technique to gain supporting information for certain scientific studies, thermal diffuse scattering (TDS) measurement is possible. TDS is performed using an X-ray beam with an energy bandwidth of 100 meV. This is achieved by translating the second pair of the HRM Si(15119) out of the beam and inserting a Si(220) channel-cut into the beam. The Si(220) channel-cut is mounted on the cryo-box of the second pair; both pairs of crystals are separated by 70 mm horizontally along the rotation axis. Both beams (1 meV and 100 meV bandwidth) have the same beam path after the HRM.

**Figure 11:**
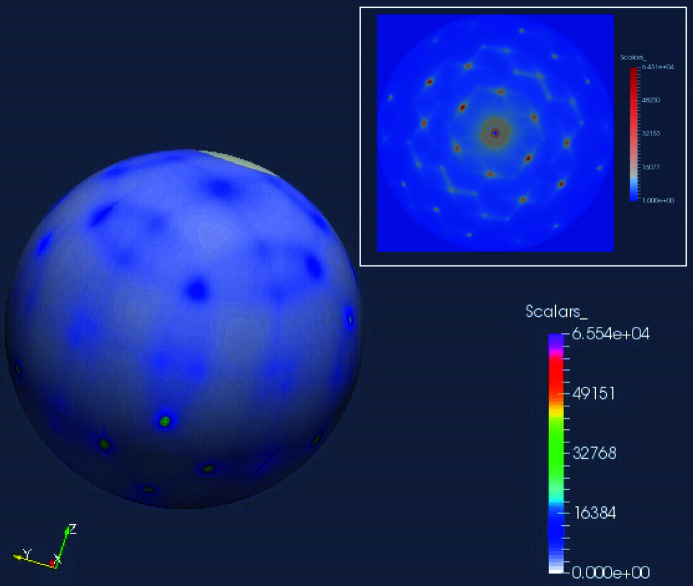
3D reciprocal space map for silicon crystal. The inset is a cross-section along Si(111).

Reciprocal space mapping is accomplished by integrating diffraction images collected at different incident angles. Fig. 11 ▸ shows one demonstration measurement on a silicon crystal. The Si crystal was rotated by 360° with a step size of 0.25°; an image was collected at each step size. The 3D reciprocal space construction is performed using the *RSMap3D* software package (Hammonds, 2016 ▸).

## 5. Sample environment   

**Figure 12:**
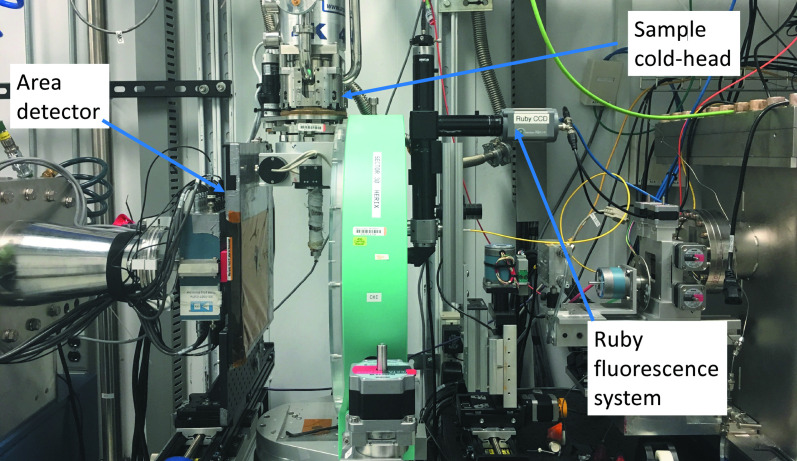
Photograph of the ruby fluorescence system, PE area detector and the sample environment.

**Figure 13:**
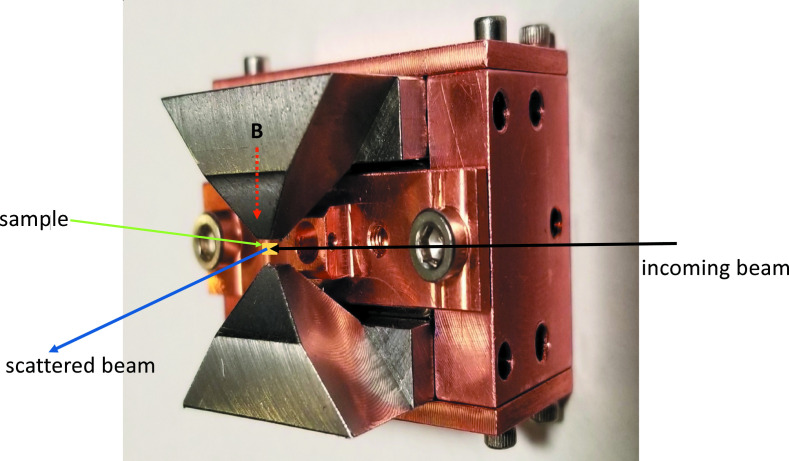
Photograph of the magnetic field apparatus.

The following sample environments are available:

(i) Low/high temperature using a closed-cycle cryostat with a temperature range of 4–800 K.

(ii) High pressure using a helium-driven membrane diamond anvil cell (DAC), which can be used at ambient and low temperatures. An *in situ* ruby fluorescence system for pressure determination is available, as shown in Fig. 12 ▸. A high-pressure DAC preparation/loading laboratory and gas loading system are available for users at the APS.

(iii) High temperature using an in-vacuum heating stage that can go up to 1000 K.

(iv) Magnetic field using a permanent magnet apparatus which can go up to 2.2 T at room temperature. The magnet can be used inside a cryostat; the magnetic field direction can be aligned to be in or out of the scattering plane (see Fig. 13 ▸).

(v) Uniaxial strain using cryogenic strain cell from Razorbill (model number CS100). The cell allows for tensile and compressive strains to samples. The maximum applicable strain displacement is 12 µm (±6 µm) at room temperature and half of the range at 10 K. The sample is pushed and pulled by piezo stacks, and the strain is measured by capacitance. More details about the cell can be found at https://razorbillinstruments.com/uniaxial-strain-cell/.

## 6. Summary   

We have described the design parameters, components and capabilities of the high-energy-resolution inelastic X-ray scattering HERIX spectrometer located at Sector 30 of the Advanced Photon Source. The HERIX spectrometer is a state-of-the-art spectrometer that provides: (i) high resolving power, 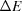/*E* ≃ 6 × 10^−8^, at *E* = 23.724 keV with flux at the sample of 4.5 × 10^9^ photons s^−1^ meV^−1^; (ii) micro-focused beam, 35 µm × 15 µm (H × V); (iii) nine analyzers; and (iv) large momentum transfer, *Q*_max_ = 72 nm^−1^. A wide range of sample environments are available covering a temperature range of 5–1000 K at ambient pressure and 5–300 K at high pressure. The plans for the HERIX instrument within the upcoming APS upgrade includes an upgrade to the HRM monochromator. All crystals of the new HRM will be operated at 123 K with an active feedback control on both temperature and crystal angles. This HRM should significantly improve energy stability based on the test results of a prototype at Sector 3 (Toellner *et al.*, 2015 ▸).

## Figures and Tables

**Table 1:** Parameters of the HERIX spectrometer at 30ID at the APS; *Q* = 2*k*sin(θ), *k* = 2π/λ

*E* (keV), λ (Å)	Energy resolution (meV)	No. of analyzers	Maximum 2θ(*Q*) degree (nm^^−1^^)	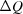 (nm^−1^)	Beam size, H × V (µm)	Flux at sample (photons s^−1^ meV^−1^)
23.724, 0.5226	1.3–1.7	9	35 (72)	±(0.1–0.7)	35 × 15	4.5 × 10^9^
